# Transcriptional profile of hippocampal dentate granule cells in four rat epilepsy models

**DOI:** 10.1038/sdata.2017.61

**Published:** 2017-05-09

**Authors:** Raymond Dingledine, Douglas A. Coulter, Brita Fritsch, Jan A. Gorter, Nadia Lelutiu, James McNamara, J. Victor Nadler, Asla Pitkänen, Michael A. Rogawski, Pate Skene, Robert S. Sloviter, Yu Wang, Wytse J. Wadman, Claude Wasterlain, Avtar Roopra

**Affiliations:** 1 Department of Pharmacology, Emory University, Atlanta, Georgia 30322, USA; 2 Department of Neurology, Children’s Hospital of Philadelphia, Philadelphia, Pennsylvania 19104, USA; 3 Department of Neurology, University Hospital Freiburg, 79106 Freiburg, Germany; 4 Swammerdam Institute for Life Science, Center for Neuroscience, University of Amsterdam, Science Park 904, Amsterdam 1098 XH, Netherlands; 5 Department of Neurobiology, Duke University, Durham, North Carolina 27710, USA; 6 A.I.Virtanen Institute for Molecular Sciences, University of Eastern Finland, PO Box 1627, Kuopio FIN-70211, Finland; 7 Departments of Neurology and Pharmacology, School of Medicine, University of California, Davis, Sacramento, California 95817, USA; 8 Department of Neurobiology, Morehouse School of Medicine, Atlanta, Georgia 30310, USA; 9 Department of Neurology and Brain Research Institute, and VA Greater Los Angeles Health Care System, Univ. California Los Angeles, Los Angeles, California 90073, USA; 10 Department of Neuroscience, Univ. Wisconsin, Madison, Wisconsin 53705, USA

**Keywords:** Transcriptomics, Epilepsy

## Abstract

Global expression profiling of neurologic or psychiatric disorders has been confounded by variability among laboratories, animal models, tissues sampled, and experimental platforms, with the result being that few genes demonstrate consistent expression changes. We attempted to minimize these confounds by pooling dentate granule cell transcriptional profiles from 164 rats in seven laboratories, using three status epilepticus (SE) epilepsy models (pilocarpine, kainate, self-sustained SE), plus amygdala kindling. In each epilepsy model, RNA was harvested from laser-captured dentate granule cells from six rats at four time points early in the process of developing epilepsy, and data were collected from two independent laboratories in each rodent model except SSSE. Hierarchical clustering of differentially-expressed transcripts in the three SE models revealed complete separation between controls and SE rats isolated 1 day after SE. However, concordance of gene expression changes in the SE models was only 26–38% between laboratories, and 4.5% among models, validating the consortium approach. Transcripts with unusually highly variable control expression across laboratories provide a ‘red herring’ list for low-powered studies.

## Background and Summary

Epileptogenesis is the process that causes networks in a normal brain to become a source of epileptic seizures. Among the numerous triggers of this process is a *de novo* bout of convulsive status epilepticus (SE)^1^, which is defined operationally as seizures lasting more than 5 min (typically >30 min), or a cluster of seizures that occur so closely together that full consciousness is not regained between seizures^2^. Both epidemiological and preclinical observations support the idea that status epilepticus (SE) at any age can result in epilepsy later in life. Between 13–82% of children who experience a febrile convulsive SE eventually develop epilepsy, with a long latent period of up to 10 years^3^, whereas about 30% of teens or adults who experience SE develop epilepsy within two years^1^. Uncertainty surrounding the mechanisms underlying epileptogenesis has limited progress towards interventions, although the extended time required in both rodents and man for clinically obvious seizures to develop suggests that gene expression changes might contribute to epileptogenesis. Numerous attempts have been made to employ transcriptional expression profiles in animal models to identify early events in epileptogenesis (e.g., refs 4–10) but, aside from transcripts pointing to an expected inflammatory state, no consensus has developed regarding common transcriptional drivers of epileptogenesis.

Discrepancies among studies likely reflect a combination of low statistical power and technical factors such as differences in the selection of species, variability in model methods, different brain regions selected for study, and the predominant cell type that was investigated. To address these issues, we assembled a group of laboratories into an ‘epilepsy microarray consortium’ to create epileptic rats using a variety of models, with the broad objective of creating a reliable dataset of transcriptional profiles in a single neuron type during the early phase of epileptogenesis. We reasoned that common transcriptional changes among the diverse models would identify changes fundamental to epileptogenesis, irrespective of the model used. We selected Sprague-Dawley rats because they are outbred and they avoid the substrain differences often found with inbred mouse strains. We laser-captured dentate granule cells to avoid the interpretive complications of pooling information from multiple cell types, to avoid changes in cellular makeup due to injury, to minimize the contribution of cell death pathways compared to analysis of dying CA1 or CA3 pyramidal neurons^11^, and because these neurons control generalization of seizures through hippocampal circuits^12–15^. Additionally, the number of differentially expressed transcripts in dentate granule cells 1 day after brief kainate-induced seizures was 4–10 times higher than in the CA1 or CA3 cell layers^16^, so the transcriptional response of these neurons to seizures is high. To minimize model-specific conclusions, four different epilepsy models were studied: SE caused by systemic pilocarpine or kainate, or electrical stimulation (SSSE); a fourth model, amygdala kindling, is not based on SE but instead relies on twice-daily 1 s duration electrical stimulation of the amygdala.

**Figure 1:**
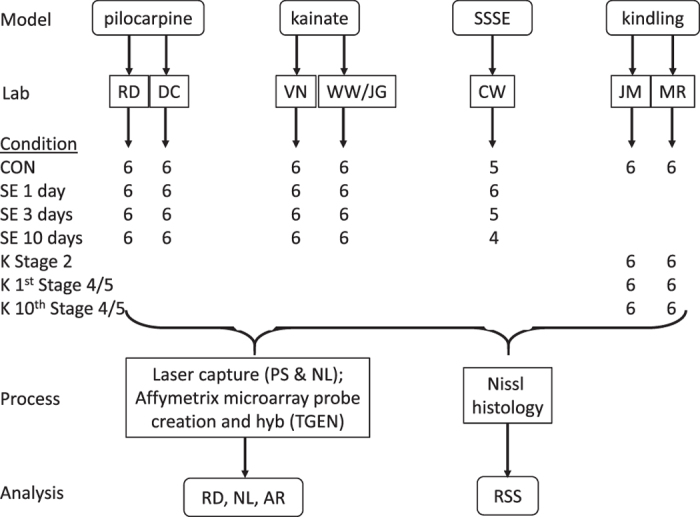
Workflow scheme. The number of rats from which high quality RNA was obtained is shown for each condition tested. Control in the case of kindling is sham stimulation. Abbreviations: RD, DC etc on the ‘Lab’, ‘Process’ or ‘Analysis’ rows are initials of the responsible authors; CON—control rats that had been injected with vehicle (pilocarpine and kainate models), or implanted but not stimulated (SSSE and kindling models); SE 1 day—rats sacrificed 1 day after experiencing status epilepticus (SE); K stage 2—rats undergoing kindling that were sacrificed 24 h after the first Stage 2 seizure. SSSE—self-sustained status epilepticus, produced by continuous electrical stimulation of the angular bundle.

Each model was studied in two independent laboratories to mitigate laboratory-to-laboratory variability. Dentate granule cells were harvested from tissue generated by each laboratory. Rats were produced at each of three time points plus controls, and hemibrains from all rats were examined histologically in a single laboratory to identify outliers that had much more (or less) neurodegeneration than expected. Figure 1 shows the study design. The initial goals of this study were to: a) identify model-independent transcriptional changes in dentate granule cells that might point to novel intervention targets for epileptogenesis, b) characterize the basal transcriptional profile of dentate granule cells, and c) identify genes that have highly variable expression. The data, deposited in NCBI GEO, are the source of several ongoing analyses (e.g., Roopra and Dingledine, in preparation; Srivastava *et al.*, in preparation), and should be a valuable resource for future efforts to understand the process of epileptogenesis based on analyses of transcriptional profiles across animal models and laboratories. Moreover, the data from untreated rats were obtained from 41 animals across seven laboratories, and thus provide a comprehensive picture of the basal transcriptional profile of dentate granule cells.

## Methods

### Overview of experimental design

We attempted to harmonize methods and, where possible, have procedures performed by a single laboratory or individual to reduce technical variability. Thus, a) each of the two paired laboratories for each animal model agreed upon the procedure to be followed; b) all tissue was sent to a single laboratory (RD), who distributed it for histology and laser capture microdissection (LCM); c) all histology was done in a blinded fashion at a single laboratory (RSS), and LCM at two laboratories (PS and RD); d) the microarray procedures were carried out at a single site (TGEN, Phoenix, AZ).

### Animal models and tissue preparation

All animal procedures were performed in accordance with NIH guidelines or EU Directive for the protection of laboratory animals, and were approved by the IACUC of each consortium members’ institution. The animals used in this study were male Sprague-Dawley rats obtained from Charles River. Individually housed animals were handled and allowed to acclimatize for at least 1 week prior to experiments, with food and water available ad libitum. Each laboratory provided tissue from 24 animals, six per group (controls and three groups post-SE or at defined kindling stages). Control animals were collected at each of the three time points.

#### Pilocarpine model (RD, DC)

Sprague-Dawley rats (180–200 g) were purchased from Charles-River (Raleigh, NC). Rats were given an injection of methylscopolamine (1 mg kg^−1^, i.p., Sigma) to minimize peripheral cholinergic effects of pilocarpine. After 30 min, animals received pilocarpine hydrochloride (300–350 mg kg^−1^ sc, Sigma) or saline. Seizures were classified according to Racine^17^ and Schauwecker and Steward^18^ with slight modifications^11^. SE was defined by continuous seizure activity consisting of stage 4 to 6 seizures. SE was terminated with pentobarbital (25 mg kg^−1^, i.p., McKesson) after 90 min of SE activity. Animals were monitored daily and given dextrose in lactate Ringer solution (~1 ml, i.p.) as needed. Control rats were given methylscopolamine and pentobarbital, but saline instead of pilocarpine.

#### Kainate model (WW/JG and VN)

Sprague Dawley rats (175–250 g) were purchased from Charles-River, Raleigh (VN) or Harlan, Netherlands (WW/JG). The Netherlands group used an initial intraperitoneal injection of 7.5 mg kg^−1^ kainic acid, followed by 5 mg kg^−1^ kainate hourly until SE induction; seizure activity was stopped with pentobarbital 90 min after SE onset. The Duke group injected rats with kainic acid (5 mg kg^−1^, i.p.) hourly until status epilepticus was achieved and then allowed SE to self-terminate after 6–8 h. All animals received 5–6 injections before SE onset. SE onset was defined by continuous stage 4 and stage 5 seizure activity. Control rats were given saline at the same schedule.

#### Self-sustained status epilepticus (SSSE) model (AP, CW)

Male Sprague-Dawley rats were purchased from Charles River at 280–300 g. The animals were implanted with a stimulating electrode in the left angular bundle (in reference to lambda AP +0.5 mm, ML +4.5 mm, depth −4.0 mm from surface of brain). Electrodes were positioned under isoflurane anesthesia (4% in O_2_), and animals were treated for post-operative pain with buprenorphine for three days following surgery. Rats were allowed to recover for 14 days before initiating SE. EEG was recorded from skull screws located over the dorsal hippocampi. The stimulation wave form consisted of 10 s trains of 20 Hz biphasic square waves delivered at 1 train per minute superimposed on continuous 2 Hz monophasic square waves. All square waves were 1 msec in duration. Animals received no treatment following stimulation of the perforant path and were included in the study if they remained in SE 10 min after the end of perforant path stimulation^19^. Intermittent stage 1–5 kindling-like seizures, and electrographic seizures that were initially nearly continuous and later became separated by periods of low-voltage activity, continued for many hours. The mean duration of SE was 8.2±2.1 h^20^. Control animals were implanted but not stimulated.

#### Kindling (JM, MR)

Male Sprague-Dawley rats, 180–200 g at time of receipt, were obtained from Charles River. One week later a bipolar electrode was stereotaxically implanted in the right amygdala under pentobarbital anesthesia (60 mg kg^−1^, i.p.), and animals allowed to recover for 5–14 days. The electrographic seizure threshold was determined by administering a 1 s train of 1 msec biphasic rectangular pulses at 60 Hz beginning at 60 μA. Additional stimulus trains increasing by 20 μA were administered at one minute intervals until an electrographic seizure lasting at least 5 s was detected on the electroencephalogram (EEG) recorded from the amygdala. Stimulation was subsequently administered at this intensity twice daily. Unstimulated control animals underwent surgical implantation of an electrode in amygdala but were not stimulated. Behavioral seizure class was scored according to Racine’s classification^17^: Stage 0, no behavioral change; Stage 1, facial clonus; Stage 2, head nodding; Stage 3, unilateral forelimb clonus; Stage 4, rearing with bilateral forelimb clonus; Stage 5, rearing and falling (loss of postural control). Datasets were generated from animals early in the kindling process (24 h after the first Stage 2 seizure), at the completion of kindling (24 h after the first Stage 4 or 5 seizure), and after kindling had been established and was stable over time (24 h after the 10th Stage 4 or 5 seizure).

#### Tissue handling

Animals that entered status epilepticus (SE) were killed for tissue harvest at 1 day (23–25 h), 3 days (70–74 h), or 10 days after SE onset. Brains were removed from kindled rats 24 h after the first stage 2 seizure, 24 h after the first stage 5 seizure or 24 h after the tenth stage 5 seizure. At each time point, the animal was decapitated following deep ether or isoflurane anesthesia. Brains were removed and longitudinally bisected. The left half of each brain was immersed in 4% (w/v) paraformaldehyde in 0.1 M sodium phosphate buffer, pH 7.4 and stored at 4 °C, while the right half was frozen on dry ice and stored at −80 °C in foil. Each consortium laboratory followed the same procedure for brain tissue preparation. All tissue was sent to one laboratory (RD) and subsequently the fixed brains were sent to RSS for Nissl stain histology and to assess the extent of damage incurred. The frozen half of the brains from the pilocarpine and kainate epilepsy models were sent to PS for laser capture microscopy of dentate granule cells. Laser capture for the brains produced by SSSE was done at Emory University. Our goal was to process tissue from 6 rats at each time point from each laboratory, although as Fig. 1 indicates, some brains had thawed in shipment and were thus unusable.

### Laser capture microdissection and RNA isolation

**Figure 2:**
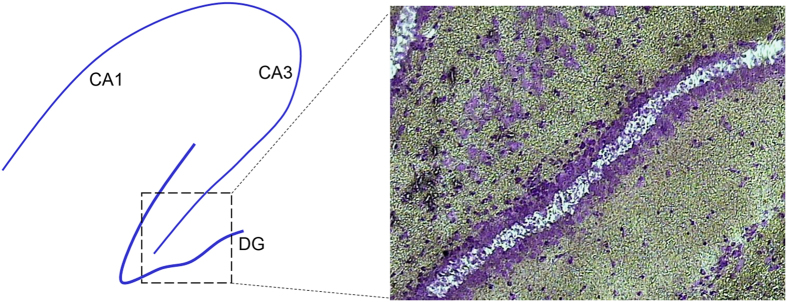
Laser capture harvesting of cells in the middle of the dentate granule cell layer in the outer leaf. Left: diagram of hippocampus with CA1 and CA3 pyramidal cell layers and the dentate gyrus (DG). Laser capture was done from the boxed region and shown in the right panel. Cresyl violet stain of a section from a rat treated with pilocarpine 10 days before. The region of captured dentate granule cells is indicated by the absence of blue stain.

Ten micron coronal sections were taken through the hippocampus and collected onto uncoated microscope slides, refrozen on dry ice, and stored at −80 ° C until staining for LCM. For staining, sections were fixed in ice-cold 70–75% ethanol for 30 s, rinsed in water 10–15 s, dipped in cresyl violet stain 10–15 s, rinsed in H2O for 10 s, then dehydrated through alcohols over 2 min to xylene. Sections were dried in a fume hood and LCM was performed within 1 hour. LCM was performed using a Veritas or Pixcell IIe system with transmission illumination (Arcturus, CA) and the following parameters: spot size=25–30 μm; power=50–65 mW; duration=1,700–3,000 μs per hit. The middle of the dentate gyrus was harvested (Fig. 2) from 10–16 sections per subject in order to avoid the neurogenic zone of the hilar-granule cell border. LCM HS Caps (Arcturus) were used to collect dentate granule cells with 2–5 sections per cap, resulting in 2–8 LCM Caps used per subject and at least 10 ng total RNA for amplification.

Total RNA was extracted from each LCM cap using the PicoPure Isolation Kit (Arcturus) with DNase digestion (Qiagen Rnase-free DNase Set), and stored at −80 °C until RNA quality verification and quantification using Agilent RNA 6,000 PicoChips run on the Agilent 2,100 Bioanalyzer. The results of the PicoChip determined whether the RNA was of high enough quality to keep for amplification and later hybridization. Total RNA with 28S:18S rRNA ratio >0.8 and RNA Integrity Number (RIN)>6.9 was accepted from each LCM cap in order to obtain one pooled sample from each rat with a minimum of 10 ng total RNA. Once a total RNA pool was complete for each subject, the sample was assessed by the BioAnalyzer, quantity and quality recorded, and samples were frozen at −80 °C until shipment to TGEN for RNA amplification and microarray hybridization.

### Microarray hybridization

The NINDS NIMH Microarray Consortium at the Translational Genomics Institute in Phoenix, AZ (TGEN) performed RNA amplification, sample labeling, microarray hybridization, and submission of the data to NCBI GEO. Briefly, TGEN amplified the RNA once, reverse-transcribed the RNA to cDNA and used this to produce biotinylated cRNA with the EnzoBioArray High Yield RNA Transcript Labeling Kit (Affymetrix, CA). Samples (10 μg) were hybridized for 16 h at 45 °C on the GeneChip Rat Genome 230 2.0 Array. GeneChips were washed and stained in the Affymetrix Fluidics Station 400. Probe creation and hybridization was done by two technicians at TGEN; JM, MR, VN and CW samples were processed by one technician, whereas RD, DC and WW samples were processed by a second technician. The RAE230A is a high-density microarray that surveys over 10,000 unique transcripts. GeneChips were scanned using the GeneArray Scanner G2500A. The data were analyzed with Microarray Suite version 5.0 (MAS 5.0) using Affymetrix default analysis settings and global scaling as normalization method. The mean target intensity of each array was set to 150. All image files and data from hybridizations are available at the NCBI GEO website, with access number GSE47752 (Data Citation 1).

### Neuropathology

Fixed half-brains from each laboratory were sent to RSS for histological assessment of cell damage and neuronal death in the hippocampus. For each brain, 40-μm-thick coronal sections were cut in 0.1 M Tris (hydroxymethylaminomethane) buffer pH 7.6, with a Vibratome. Regularly spaced sections were mounted on subbed slides for subsequent Nissl (1% cresyl violet) staining. After staining, all slides were dehydrated in graded ethanols and xylene and then coverslipped with Permount^21^. All tissue from SE-induced animals was assessed against that of controls for cell damage in various areas of the hippocampus, including the CA1, CA3, hilus, dentate gyrus, and piriform cortex. An outlier of SE-brains was defined as either a) one with no discernable neuron death in CA3 or CA1, or b) a brain with an extremely injured hippocampus that includes substantial granule cell death. No outliers were found, so data from all rats were included in the analysis.

### Microarray data processing

Affymetrix CEL and Rat230_2.cdf files were used for RMA normalization with background adjust, quantile normalization and median polish. Annotation was performed using netaffx build 35. RMA normalization was performed using all probes regardless of Affymetrix ‘Present’ (P) or ‘Absent’ (A) calls. Subsequently, only probes with the ‘_at’ suffix were retained to ensure that only probes specific to a single gene were carried forward for further analysis. Probe level data were then collapsed to gene symbols with the symbol being designated ‘Present’ in a RNA sample if at least 50% of probes for that symbol were designated as ‘Present’. To designate genes as expressed or not over the entire experiment the following rules were applied: A symbol was designated ‘above background’ in a laboratory for a given status epilepticus time point and epilepsy model (e.g., Day 1 after pilocarpine SE, see Fig. 1) if it was labelled ‘Present’ in at least 3 of the animals at that time point. A gene symbol was designated as ‘above background’ for a specific time point if it was labelled ‘Present’ in at least 4 of the 5 SE laboratories (i.e., no more than 1 lab could call the symbol ‘Absent’). For the control condition, the symbol was considered above background if it was called Present in at least 6 of the 7 laboratories (i.e., no more than 1 laboratory could call the symbol ‘Absent’). Finally, a symbol was designated ‘above background’ over the entire analysis if it was called as Present in any time point. For each gene symbol accepted for the entire analysis, expression values for every animal were included regardless of its Absent or Present call in any individual animal. In summary: A symbol is expressed above background in a laboratory at a given time point if it is labeled as Present in at least 3 animals. A symbol is above background in SE if it is Present in at least 4 laboratories. For controls, the symbol is above background if it is labeled Present in at least 6 laboratories.

To cluster expression values pooled across SE models and laboratories, the expression level of each gene symbol in a laboratory was collapsed to its median log2 value across animals in that laboratory. A total of 9,614 symbols were Present in at least 1 of the 4 conditions (control, 1- day, 3-days, 10-days after SE). Paired *t*-tests compared expression levels after SE with control values and *n*=5 labs, then a correction^22^ was made for multiple comparisons to adjust the false discovery rate (FDR) to 0.05. Altogether there were 2,646 differentially expressed genes after SE at any of the three time points examined in this analysis. Unsupervised hierarchical clustering was performed using median values of the 368 genes that were differentially expressed at least 2-fold between controls and SE rats, with uncentered Pearson correlations and complete clustering. To determine the degree of overlap between differentially expressed genes 1 day after SE among the three models, unpaired *t*-tests were run separately on data from each of the 5 laboratories (FDR<0.05)^22^ and a Venn diagram approach used.

## Data Records

CEL and CHIP files associated with this analysis are deposited in the Gene Expression Omnibus (https://www.ncbi.nlm.nih.gov/gds/) with the accession number GSE47752 (Data Citation 1). Detailed information about each sample is in Table 1 (available online only).

## Technical Validation

Several aspects of the experiment were designed to improve the quality of the data. First, RNA was harvested from the middle of the dentate granule cell layer (Fig. 2) both to minimize contributions from multiple cell types that would have occurred had we used whole tissue (e.g., hippocampus) instead, and to avoid contamination by cells undergoing neurogenesis at the hilar-granule cell border. However, low to moderate levels of RNA from glial processes found in the cell layer are included; e.g., the microglial transcript that encodes Iba1 is expressed at a level of 104, the oligodendrocyte marker MBP is 612 and the astrocytic marker GFAP is 2,219. Second, we gathered data in parallel from 4 epilepsy models to provide a database from which model-independent conclusions can be drawn. Third, we intended that two independent laboratories study each animal model to reduce lab-to-lab influences; this was successful except for the SSSE model as described above. Fourth, 6 rats (occasionally 4–5) were provided for each treatment group from each laboratory; e.g., the control group was derived from 41 rats in 7 laboratories. Fifth, to minimize technical variation the LCM harvest of RNA from 144 of the 164 samples was done in one laboratory by the same technician, and the remainder (the SSSE samples) in a second laboratory; likewise RNA amplification, hybridization probe generation, microarray hybridization and initial data reduction were done at a single site (TGEN in Phoenix, AZ) by two technicians. Finally, RNA quality was controlled by accepting pooled samples from a rat if the RIN≥6.9, the ratio of 28S to 18S rRNA was ≥0.8, and at least 10 ng of total RNA had been obtained from the granule cells.

**Figure 3:**
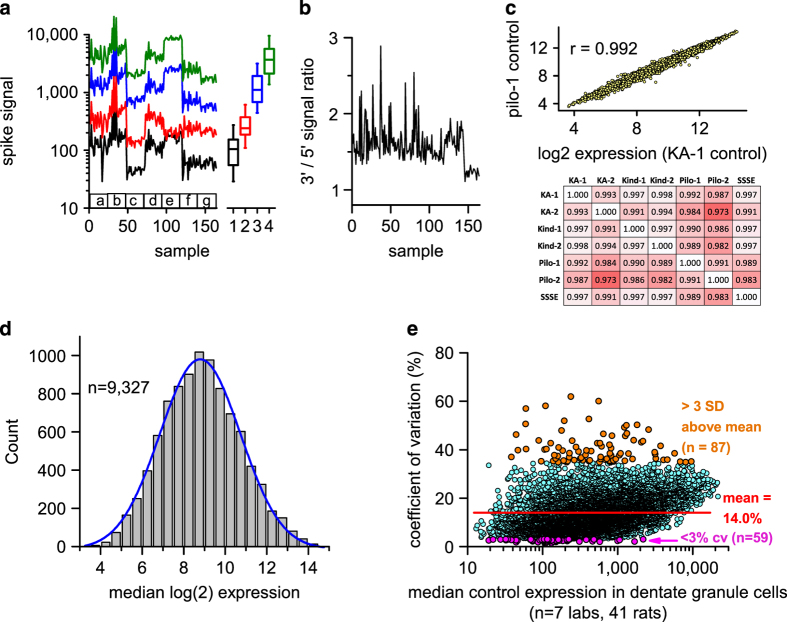
Quality control assessment for microarrays of the RNA samples from 164 rats. (**a**) Signal values from 4 bacterial or viral RNAs that had been spiked into each sample RNA before probe labeling are plotted for each of the 164 RNA samples. Boxplots of median expression across all arrays are shown to the right. Labels a-g denote data from the seven laboratories. Labels 1–4 are different prokaryotic or phage RNAs spiked at increasing levels. (**b**) Median 3′/5′ intensity ratios from 10 transcripts in each sample are plotted. (**c**) Excellent correlation between expression in the control condition of the 9,327 transcripts in the first kainate laboratory (KA-1) and the first pilocarpine laboratory (pilo-1). The heatmap below shows Pearson’s correlations between median control expression in all combinations of the 7 laboratories; correlations range from 0.973 to 0.998. (**d**) Histogram of the median expression level of each expressed gene in the control condition across all 7 laboratories is normal (Kolmogorov-Smirnov test). (**e**) The coefficient of variation is plotted versus the median expression for each of 9,327 transcripts in the control condition across 7 laboratories, revealing a subset of 59 genes with very tight expression across laboratories (<3% cv) and a subset of 87 genes with very variable expression. (**c**–**e**): A transcript was selected for inclusion if at least 6 of the 7 laboratories had no more than 3 absent calls each (mean of 0.26 absent calls/gene/lab).

To explore the technical quality and uniformity of the raw microarray data, we carried out two diagnostic tests. Into each RNA sample Affymetrix routinely spikes RNAs from three E. coli genes (bioB, bioC, bioD) and the bacteriophage cre recombinase to assess the overall success of probe synthesis, hybridization and microarray imaging. We plotted the array signals from each of these 4 spikes over the 164 rat samples in Fig. 3a. Success requires that signal intensities from these 4 spikes show increasing values. The associated boxplots of median expression of these spikes demonstrate that the 4 RNA spikes are present at the expected increasing levels. Importantly, the lowest level spike was called ‘present’ in all 164 samples. Second, we plotted the median ratio of signal intensities for probes hybridizing to the 3’ and 5’ regions of 7 spiked RNAs and 3 endogenous RNAs to assess RNA degradation during the process. Figure 3b shows that all 164 samples had a 3’/5’ ratios<3, indicative of acceptable RNA quality.

To further examine the quality of the data and the consequences of our experimental design we carried out several additional tests on data that were filtered as described above. To determine variation of expression levels across laboratories we considered the 41 controls provided by all 7 laboratories. The samples provided by each lab showed high correlation of signal intensities over the 9,327 transcripts studied (Fig. 3c). Pearson correlation coefficients ranged from 0.973 to 0.998 as shown in the heatmap in Fig. 3c. The median expression intensities of each transcript were then calculated across the 7 laboratories, with each laboratory donating a single expression value for each transcript derived from the median of all control rats. A histogram of median log2 values of expression in the control condition across the 7 sites was normally distributed (Fig. 3d), with no ‘excess’ representation of low-expressing noise transcripts. A plot of the coefficient of variation of expression level across laboratories for each of the 9,237 transcripts versus its median expression level (Fig. 3e) showed that the mean CV of expression was 14%. Moreover, this plot revealed the presence of transcripts with highly stable expression across sites (*n*=59 with CV<3%, see Table 2 (available online only)), and transcripts with highly variable expression (*n*=87 with CV>3 SDs above the mean, see Table 3 (available online only)). The high variability group of transcripts represent potential ‘red herrings’ in transcriptional profiling studies that have low power.

**Figure 4:**
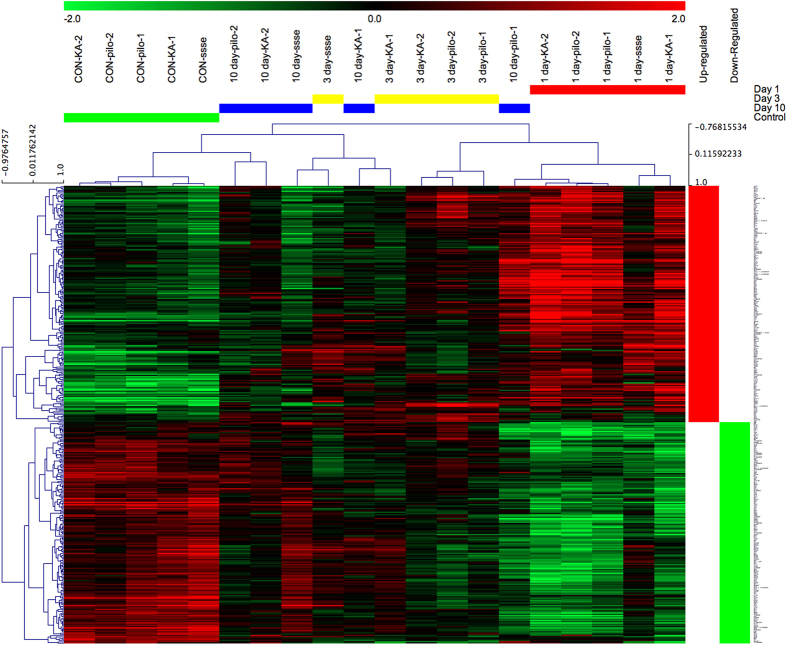
Hierarchical clustering of differentially expressed genes across laboratories and SE epilepsy models. Unsupervised hierarchical clustering was performed using median log2 values of the 368 genes that were differentially expressed (FDR<0.05) at least 2-fold between controls and SE-experienced rats, in any model and any lab, with uncentered Pearson correlations and complete clustering.

**Figure 5:**
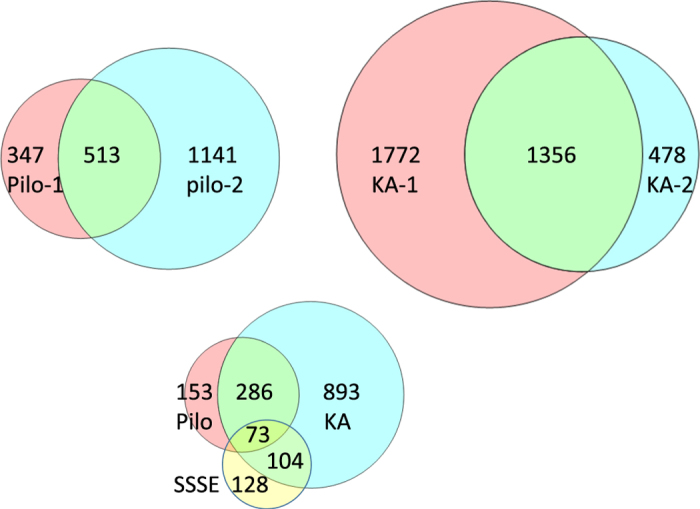
Low congruence of differentially-expressed genes across laboratories and animal models of epilepsy 1 day after SE. The circles in the top row show overlap between laboratories studying the same epilepsy model, and the bottom compares differentially expressed genes among the three epilepsy models.

To determine whether our strategy of comparing animal models and of having two independent laboratories study each animal model was worthwhile, we focused on the SE models for which we have the most data. We first performed a hierarchical clustering of expression levels across all four time points and three SE models (control and 1, 3 or 10 days after SE), with data from all rats in each group from each laboratory having been condensed to their median expression level for each transcript. Figure 4 shows complete separation of the 5 control groups and the 5 SE groups 1 day after SE, whereas profiles from the 3 day and 10 day groups were somewhat intermixed. This suggests that the datasets are sufficiently robust at the group level to distinguish SE from control rats reliably in all laboratories, which is a prerequisite for future studies of the whole dataset. Second, to determine whether our experimental design incorporating multiple laboratories to study each animal model was necessary, we used a Venn diagram approach to compare transcript levels 1 day after SE in the pilocarpine, kainate and SSSE models. We found low congruence between paired laboratories and especially among the three models (Fig. 5). Very few transcripts were differentially expressed in any of the kindling groups, and none of those overlapped with the SE groups. These results validate the importance of a consortium strategy for the goal of identifying the relatively small number of transcripts that are differentially expressed in a laboratory- and model-independent fashion. Thus, 1 day after SE only 73 out of 1,638 transcripts differentially expressed in any model in any laboratory (4.5%) were in common across all laboratories and models. These transcripts (Table 4 (available online only)) represent genes whose differential expression in response to SE was independently replicated for each model and laboratory combination. The large group of transcripts that do not overlap in the Venn diagrams may reflect expression changes unique to each treatment model or laboratory. The results suggest that all three SE models converge at the one day time point on a rather small common set of gene expression changes associated with the early development of epilepsy, or potentially with nearby brain injury. Our dataset of transcripts with the most reproducible changes in expression (Table 4 (available online only)) should be useful for numerous analyses of seizure-induced gene expression changes in the rat. None of these transcripts appears in Tables 2 and 3 (available online only).

The deposited data on transcriptional responses of dentate granule cells to SE 1, 3 and 10 days later, and throughout the kindling process, provide a reference dataset that can be extended in multiple ways. It would be useful to compare dentate granule cells with other cell types thought to be involved in epileptogenesis, for example the principal neurons in layer II of the medial entorhinal cortex^23^ or astrocytes^24,25^. It would also be worthwhile to examine transcriptional responses earlier in the process (e.g., 1–6 h after SE onset), during which neurogenic inflammation is initiated^26^. Finally, other epilepsy models such as febrile SE^27^, post-traumatic epilepsy^28^ or genetic epilepsies that do not involve overt neurodegeneration^29^, could be considered. The genetic epilepsies might avoid transcriptional changes due to a bystander effect of nearby dying neurons; mice bearing epilepsy genes with late expression onset would lend themselves to the kind of temporal analysis done here but could suffer from effects on development unrelated to epileptogenesis. The cost of RNAseq analyses has now ebbed to the point at which it could be used in future studies and would open the analysis to splicing isoforms of individual transcripts and to non-coding RNAs e.g., miRNAs and lncRNAs. Regardless how this study is extended, it is worth recognizing the value shown here of utilizing multiple models and multiple investigators, and harvesting RNA from single cell types to limit noise in the transcriptome.

## Additional Information

**How to cite this article:** Dingledine, R. *et al.* Transcriptional profile of hippocampal dentate granule cells in four rat epilepsy models. *Sci. Data* 4:170061 doi: 10.1038/sdata.2017.61 (2017).

**Publisher’s note:** Springer Nature remains neutral with regard to jurisdictional claims in published maps and institutional affiliations.

## Supplementary Material



## Figures and Tables

**Table 1:** Sample description

**GEO-ID**	**organism**	**tissue**	**sample description**	**analysis**
GSM1155788	Rattus norvegicus albino	dentate granule neurons	kainate-Nadler-control-rat 1	microarray
GSM1155864	Rattus norvegicus albino	dentate granule neurons	kainate-Nadler-control-rat 2	microarray
GSM1155878	Rattus norvegicus albino	dentate granule neurons	kainate-Nadler-control-rat 3	microarray
GSM1155804	Rattus norvegicus albino	dentate granule neurons	kainate-Nadler-control-rat 4	microarray
GSM1155820	Rattus norvegicus albino	dentate granule neurons	kainate-Nadler-control-rat 5	microarray
GSM1155841	Rattus norvegicus albino	dentate granule neurons	kainate-Nadler-control-rat 6	microarray
GSM1155858	Rattus norvegicus albino	dentate granule neurons	kainate-Nadler-day 10-rat 1	microarray
GSM1155872	Rattus norvegicus albino	dentate granule neurons	kainate-Nadler-day 10-rat 2	microarray
GSM1155797	Rattus norvegicus albino	dentate granule neurons	kainate-Nadler-day 10-rat 3	microarray
GSM1155814	Rattus norvegicus albino	dentate granule neurons	kainate-Nadler-day 10-rat 4	microarray
GSM1155835	Rattus norvegicus albino	dentate granule neurons	kainate-Nadler-day 10-rat 5	microarray
GSM1155850	Rattus norvegicus albino	dentate granule neurons	kainate-Nadler-day 10-rat 6	microarray
GSM1155832	Rattus norvegicus albino	dentate granule neurons	kainate-Nadler-day 1-rat 1	microarray
GSM1155865	Rattus norvegicus albino	dentate granule neurons	kainate-Nadler-day 1-rat 2	microarray
GSM1155789	Rattus norvegicus albino	dentate granule neurons	kainate-Nadler-day 1-rat 3	microarray
GSM1155806	Rattus norvegicus albino	dentate granule neurons	kainate-Nadler-day 1-rat 4	microarray
GSM1155822	Rattus norvegicus albino	dentate granule neurons	kainate-Nadler-day 1-rat 5	microarray
GSM1155843	Rattus norvegicus albino	dentate granule neurons	kainate-Nadler-day 1-rat 6	microarray
GSM1155793	Rattus norvegicus albino	dentate granule neurons	kainate-Nadler-day 3 -rat 3	microarray
GSM1155854	Rattus norvegicus albino	dentate granule neurons	kainate-Nadler-day 3-rat 1	microarray
GSM1155868	Rattus norvegicus albino	dentate granule neurons	kainate-Nadler-day 3-rat 2	microarray
GSM1155810	Rattus norvegicus albino	dentate granule neurons	kainate-Nadler-day 3-rat 4	microarray
GSM1155826	Rattus norvegicus albino	dentate granule neurons	kainate-Nadler-day 3-rat 5 rehyb	microarray
GSM1155846	Rattus norvegicus albino	dentate granule neurons	kainate-Nadler-day 3-rat 6	microarray
GSM1155897	Rattus norvegicus albino	dentate granule neurons	kainate-Wadman-control-rat 1	microarray
GSM1155898	Rattus norvegicus albino	dentate granule neurons	kainate-Wadman-control-rat 2	microarray
GSM1155899	Rattus norvegicus albino	dentate granule neurons	kainate-Wadman-control-rat 3	microarray
GSM1155900	Rattus norvegicus albino	dentate granule neurons	kainate-Wadman-control-rat 4	microarray
GSM1155901	Rattus norvegicus albino	dentate granule neurons	kainate-Wadman-control-rat 5	microarray
GSM1155902	Rattus norvegicus albino	dentate granule neurons	kainate-Wadman-control-rat 6	microarray
GSM1155903	Rattus norvegicus albino	dentate granule neurons	kainate-Wadman-day 10-rat 1	microarray
GSM1155904	Rattus norvegicus albino	dentate granule neurons	kainate-Wadman-day 10-rat 2	microarray
GSM1155905	Rattus norvegicus albino	dentate granule neurons	kainate-Wadman-day 10-rat 3	microarray
GSM1155906	Rattus norvegicus albino	dentate granule neurons	kainate-Wadman-day 10-rat 4	microarray
GSM1155907	Rattus norvegicus albino	dentate granule neurons	kainate-Wadman-day 10-rat 5	microarray
GSM1155908	Rattus norvegicus albino	dentate granule neurons	kainate-Wadman-day 10-rat 6	microarray
GSM1155909	Rattus norvegicus albino	dentate granule neurons	kainate-Wadman-day 1-rat 1	microarray
GSM1155910	Rattus norvegicus albino	dentate granule neurons	kainate-Wadman-day 1-rat 2	microarray
GSM1155911	Rattus norvegicus albino	dentate granule neurons	kainate-Wadman-day 1-rat 3	microarray
GSM1155912	Rattus norvegicus albino	dentate granule neurons	kainate-Wadman-day 1-rat 4	microarray
GSM1155913	Rattus norvegicus albino	dentate granule neurons	kainate-Wadman-day 1-rat 5	microarray
GSM1155914	Rattus norvegicus albino	dentate granule neurons	kainate-Wadman-day 1-rat 6	microarray
GSM1155915	Rattus norvegicus albino	dentate granule neurons	kainate-Wadman-day 3-rat 1	microarray
GSM1155916	Rattus norvegicus albino	dentate granule neurons	kainate-Wadman-day 3-rat 2	microarray
GSM1155917	Rattus norvegicus albino	dentate granule neurons	kainate-Wadman-day 3-rat 3	microarray
GSM1155918	Rattus norvegicus albino	dentate granule neurons	kainate-Wadman-day 3-rat 4	microarray
GSM1155919	Rattus norvegicus albino	dentate granule neurons	kainate-Wadman-day 3-rat 5	microarray
GSM1155920	Rattus norvegicus albino	dentate granule neurons	kainate-Wadman-day 3-rat 6	microarray
GSM1155786	Rattus norvegicus albino	dentate granule neurons	kindling-McNamara-control-rat 1	microarray
GSM1155862	Rattus norvegicus albino	dentate granule neurons	kindling-McNamara-control-rat 2	microarray
GSM1155876	Rattus norvegicus albino	dentate granule neurons	kindling-McNamara-control-rat 3	microarray
GSM1155802	Rattus norvegicus albino	dentate granule neurons	kindling-McNamara-control-rat 4	microarray
GSM1155818	Rattus norvegicus albino	dentate granule neurons	kindling-McNamara-control-rat 5	microarray
GSM1155839	Rattus norvegicus albino	dentate granule neurons	kindling-McNamara-control-rat 6	microarray
GSM1155833	Rattus norvegicus albino	dentate granule neurons	kindling-McNamara-stage 2-rat 1	microarray
GSM1155866	Rattus norvegicus albino	dentate granule neurons	kindling-McNamara-stage 2-rat 2	microarray
GSM1155790	Rattus norvegicus albino	dentate granule neurons	kindling-McNamara-stage 2-rat 3	microarray
GSM1155807	Rattus norvegicus albino	dentate granule neurons	kindling-McNamara-stage 2-rat 4	microarray
GSM1155823	Rattus norvegicus albino	dentate granule neurons	kindling-McNamara-stage 2-rat 5	microarray
GSM1155844	Rattus norvegicus albino	dentate granule neurons	kindling-McNamara-stage 2-rat 6	microarray
GSM1155855	Rattus norvegicus albino	dentate granule neurons	kindling-McNamara-first stage 4/5-rat 1	microarray
GSM1155869	Rattus norvegicus albino	dentate granule neurons	kindling-McNamara-first stage 4/5-rat 2	microarray
GSM1155794	Rattus norvegicus albino	dentate granule neurons	kindling-McNamara-first stage 4/5-rat 3	microarray
GSM1155811	Rattus norvegicus albino	dentate granule neurons	kindling-McNamara-first stage 4/5-rat 4	microarray
GSM1155828	Rattus norvegicus albino	dentate granule neurons	kindling-McNamara-first stage 4/5-rat 5	microarray
GSM1155847	Rattus norvegicus albino	dentate granule neurons	kindling-McNamara-first stage 4/5-rat 6a	microarray
GSM1155859	Rattus norvegicus albino	dentate granule neurons	kindling-McNamara-10th stage 4/5-rat 1	microarray
GSM1155873	Rattus norvegicus albino	dentate granule neurons	kindling-McNamara-10th stage 4/5-rat 2	microarray
GSM1155798	Rattus norvegicus albino	dentate granule neurons	kindling-McNamara-10th stage 4/5-rat 3	microarray
GSM1155815	Rattus norvegicus albino	dentate granule neurons	kindling-McNamara-10th stage 4/5-rat 4	microarray
GSM1155836	Rattus norvegicus albino	dentate granule neurons	kindling-McNamara-10th stage 4/5-rat 5	microarray
GSM1155851	Rattus norvegicus albino	dentate granule neurons	kindling-McNamara-10th stage 4/5-rat 6	microarray
GSM1155787	Rattus norvegicus albino	dentate granule neurons	kindling-Rogawski-control-rat 1	microarray
GSM1155863	Rattus norvegicus albino	dentate granule neurons	kindling-Rogawski-control-rat 2	microarray
GSM1155877	Rattus norvegicus albino	dentate granule neurons	kindling-Rogawski-control-rat 3	microarray
GSM1155803	Rattus norvegicus albino	dentate granule neurons	kindling-Rogawski-control-rat 4	microarray
GSM1155819	Rattus norvegicus albino	dentate granule neurons	kindling-Rogawski-control-rat 5	microarray
GSM1155840	Rattus norvegicus albino	dentate granule neurons	kindling-Rogawski-control-rat 6	microarray
GSM1155834	Rattus norvegicus albino	dentate granule neurons	kindling-Rogawski-stage 2-rat 1	microarray
GSM1155867	Rattus norvegicus albino	dentate granule neurons	kindling-Rogawski-stage 2-rat 2	microarray
GSM1155791	Rattus norvegicus albino	dentate granule neurons	kindling-Rogawski-stage 2-rat 3	microarray
GSM1155808	Rattus norvegicus albino	dentate granule neurons	kindling-Rogawski-stage 2-rat 4	microarray
GSM1155824	Rattus norvegicus albino	dentate granule neurons	kindling-Rogawski-stage 2-rat 5	microarray
GSM1155845	Rattus norvegicus albino	dentate granule neurons	kindling-Rogawski-stage 2-rat 6	microarray
GSM1155856	Rattus norvegicus albino	dentate granule neurons	kindling-Rogawski--first stage 4/5-rat 1	microarray
GSM1155870	Rattus norvegicus albino	dentate granule neurons	kindling-Rogawski--first stage 4/5-rat 2	microarray
GSM1155795	Rattus norvegicus albino	dentate granule neurons	kindling-Rogawski--first stage 4/5-rat 3	microarray
GSM1155812	Rattus norvegicus albino	dentate granule neurons	kindling-Rogawski--first stage 4/5-rat 4	microarray
GSM1155829	Rattus norvegicus albino	dentate granule neurons	kindling-Rogawski--first stage 4/5-rat 5	microarray
GSM1155849	Rattus norvegicus albino	dentate granule neurons	kindling-Rogawski--first stage 4/5-rat 6	microarray
GSM1155860	Rattus norvegicus albino	dentate granule neurons	kindling-Rogawski-10th stage 4/5-rat 1	microarray
GSM1155874	Rattus norvegicus albino	dentate granule neurons	kindling-Rogawski-10th stage 4/5-rat 2	microarray
GSM1155799	Rattus norvegicus albino	dentate granule neurons	kindling-Rogawski-10th stage 4/5-rat 3	microarray
GSM1155816	Rattus norvegicus albino	dentate granule neurons	kindling-Rogawski-10th stage 4/5-rat 4	microarray
GSM1155837	Rattus norvegicus albino	dentate granule neurons	kindling-Rogawski-10th stage 4/5-rat 5	microarray
GSM1155852	Rattus norvegicus albino	dentate granule neurons	kindling-Rogawski-10th stage 4/5-rat 6	microarray
GSM1155880	Rattus norvegicus albino	dentate granule neurons	pilo-Coulter-control-rat 1	microarray
GSM1155881	Rattus norvegicus albino	dentate granule neurons	pilo-Coulter-control-rat 2	microarray
GSM1155882	Rattus norvegicus albino	dentate granule neurons	pilo-Coulter-control-rat 3	microarray
GSM1155883	Rattus norvegicus albino	dentate granule neurons	pilo-Coulter-control-rat 4	microarray
GSM1155884	Rattus norvegicus albino	dentate granule neurons	pilo-Coulter-control-rat 5	microarray
GSM1155885	Rattus norvegicus albino	dentate granule neurons	pilo-Coulter-control-rat 6	microarray
GSM1155921	Rattus norvegicus albino	dentate granule neurons	pilo-Coulter-day 10-rat 1	microarray
GSM1155922	Rattus norvegicus albino	dentate granule neurons	pilo-Coulter-day 10-rat 2	microarray
GSM1155923	Rattus norvegicus albino	dentate granule neurons	pilo-Coulter-day 10-rat 3	microarray
GSM1155924	Rattus norvegicus albino	dentate granule neurons	pilo-Coulter-day 10-rat 4	microarray
GSM1155925	Rattus norvegicus albino	dentate granule neurons	pilo-Coulter-day 10-rat 5	microarray
GSM1155926	Rattus norvegicus albino	dentate granule neurons	pilo-Coulter-day 10-rat 6	microarray
GSM1155927	Rattus norvegicus albino	dentate granule neurons	pilo-Coulter-day 1-rat 1	microarray
GSM1155928	Rattus norvegicus albino	dentate granule neurons	pilo-Coulter-day 1-rat 2	microarray
GSM1155929	Rattus norvegicus albino	dentate granule neurons	pilo-Coulter-day 1-rat 3	microarray
GSM1155930	Rattus norvegicus albino	dentate granule neurons	pilo-Coulter-day 1-rat 4	microarray
GSM1155931	Rattus norvegicus albino	dentate granule neurons	pilo-Coulter-day 1-rat 5	microarray
GSM1155932	Rattus norvegicus albino	dentate granule neurons	pilo-Coulter-day 1-rat 6	microarray
GSM1155933	Rattus norvegicus albino	dentate granule neurons	pilo-Coulter-day 3-rat 1	microarray
GSM1155934	Rattus norvegicus albino	dentate granule neurons	pilo-Coulter-day 3-rat 2	microarray
GSM1155935	Rattus norvegicus albino	dentate granule neurons	pilo-Coulter-day 3-rat 3	microarray
GSM1155936	Rattus norvegicus albino	dentate granule neurons	pilo-Coulter-day 3-rat 4	microarray
GSM1155937	Rattus norvegicus albino	dentate granule neurons	pilo-Coulter-day 3-rat 5	microarray
GSM1155938	Rattus norvegicus albino	dentate granule neurons	pilo-Coulter-day 3-rat 6	microarray
GSM1155887	Rattus norvegicus albino	dentate granule neurons	pilo-Dingledine-control-rat 1a	microarray
GSM1155889	Rattus norvegicus albino	dentate granule neurons	pilo-Dingledine-control-rat 2a	microarray
GSM1155891	Rattus norvegicus albino	dentate granule neurons	pilo-Dingledine-control-rat 3a	microarray
GSM1155893	Rattus norvegicus albino	dentate granule neurons	pilo-Dingledine-control-rat 4a	microarray
GSM1155895	Rattus norvegicus albino	dentate granule neurons	pilo-Dingledine-control-rat 5a	microarray
GSM1155896	Rattus norvegicus albino	dentate granule neurons	pilo-Dingledine-control-rat 6	microarray
GSM1155939	Rattus norvegicus albino	dentate granule neurons	pilo-Dingledine-day 10-rat 1	microarray
GSM1155940	Rattus norvegicus albino	dentate granule neurons	pilo-Dingledine-day 10-rat 2	microarray
GSM1155941	Rattus norvegicus albino	dentate granule neurons	pilo-Dingledine-day 10-rat 3	microarray
GSM1155942	Rattus norvegicus albino	dentate granule neurons	pilo-Dingledine-day 10-rat 4	microarray
GSM1155943	Rattus norvegicus albino	dentate granule neurons	pilo-Dingledine-day 10-rat 5	microarray
GSM1155944	Rattus norvegicus albino	dentate granule neurons	pilo-Dingledine-day 10-rat 6	microarray
GSM1155945	Rattus norvegicus albino	dentate granule neurons	pilo-Dingledine-day 1-rat 1	microarray
GSM1155946	Rattus norvegicus albino	dentate granule neurons	pilo-Dingledine-day 1-rat 2	microarray
GSM1155947	Rattus norvegicus albino	dentate granule neurons	pilo-Dingledine-day 1-rat 3	microarray
GSM1155948	Rattus norvegicus albino	dentate granule neurons	pilo-Dingledine-day 1-rat 4	microarray
GSM1155949	Rattus norvegicus albino	dentate granule neurons	pilo-Dingledine-day 1-rat 5	microarray
GSM1155950	Rattus norvegicus albino	dentate granule neurons	pilo-Dingledine-day 1-rat 6	microarray
GSM1155951	Rattus norvegicus albino	dentate granule neurons	pilo-Dingledine-day 3-rat 1	microarray
GSM1155952	Rattus norvegicus albino	dentate granule neurons	pilo-Dingledine-day 3-rat 2	microarray
GSM1155953	Rattus norvegicus albino	dentate granule neurons	pilo-Dingledine-day 3-rat 3	microarray
GSM1155954	Rattus norvegicus albino	dentate granule neurons	pilo-Dingledine-day 3-rat 4	microarray
GSM1155955	Rattus norvegicus albino	dentate granule neurons	pilo-Dingledine-day 3-rat 5	microarray
GSM1155956	Rattus norvegicus albino	dentate granule neurons	pilo-Dingledine-day 3-rat 6	microarray
GSM1155785	Rattus norvegicus albino	dentate granule neurons	SSSE-Wasterlain-control-rat 1	microarray
GSM1155831	Rattus norvegicus albino	dentate granule neurons	SSSE-Wasterlain-control-rat 2	microarray
GSM1155853	Rattus norvegicus albino	dentate granule neurons	SSSE-Wasterlain-control-rat 3	microarray
GSM1155857	Rattus norvegicus albino	dentate granule neurons	SSSE-Wasterlain-control-rat 4	microarray
GSM1155861	Rattus norvegicus albino	dentate granule neurons	SSSE-Wasterlain-control-rat 6	microarray
GSM1155825	Rattus norvegicus albino	dentate granule neurons	SSSE-Wasterlain-day 10-rat 1	microarray
GSM1155830	Rattus norvegicus albino	dentate granule neurons	SSSE-Wasterlain-day 10-rat 2	microarray
GSM1155838	Rattus norvegicus albino	dentate granule neurons	SSSE-Wasterlain-day 10-rat 3	microarray
GSM1155842	Rattus norvegicus albino	dentate granule neurons	SSSE-Wasterlain-day 10-rat 6	microarray
GSM1155871	Rattus norvegicus albino	dentate granule neurons	SSSE-Wasterlain-day 1-rat 1	microarray
GSM1155875	Rattus norvegicus albino	dentate granule neurons	SSSE-Wasterlain-day 1-rat 2	microarray
GSM1155879	Rattus norvegicus albino	dentate granule neurons	SSSE-Wasterlain-day 1-rat 3	microarray
GSM1155792	Rattus norvegicus albino	dentate granule neurons	SSSE-Wasterlain-day 1-rat 4	microarray
GSM1155796	Rattus norvegicus albino	dentate granule neurons	SSSE-Wasterlain-day 1-rat 5	microarray
GSM1155800	Rattus norvegicus albino	dentate granule neurons	SSSE-Wasterlain-day 1-rat 6 rehyb	microarray
GSM1155805	Rattus norvegicus albino	dentate granule neurons	SSSE-Wasterlain-day 3-rat 2	microarray
GSM1155809	Rattus norvegicus albino	dentate granule neurons	SSSE-Wasterlain-day 3-rat 3	microarray
GSM1155813	Rattus norvegicus albino	dentate granule neurons	SSSE-Wasterlain-day 3-rat 4	microarray
GSM1155817	Rattus norvegicus albino	dentate granule neurons	SSSE-Wasterlain-day 3-rat 5	microarray
GSM1155821	Rattus norvegicus albino	dentate granule neurons	SSSE-Wasterlain-day 3-rat 6	microarray
				
Unused samples (preliminary hybridizations or those that failed Affymetrix Q/C)				
GSM1155827	Rattus norvegicus albino	dentate granule neurons	kainate-Nadler-day 3-rat 5	microarray
GSM1155848	Rattus norvegicus albino	dentate granule neurons	kindling-McNamara-stage 4-rat 6	microarray
GSM1155886	Rattus norvegicus albino	dentate granule neurons	pilo-Dingledine-control-rat 1	microarray
GSM1155888	Rattus norvegicus albino	dentate granule neurons	pilo-Dingledine-control-rat 2	microarray
GSM1155890	Rattus norvegicus albino	dentate granule neurons	pilo-Dingledine-control-rat 3	microarray
GSM1155892	Rattus norvegicus albino	dentate granule neurons	pilo-Dingledine-control-rat 4	microarray
GSM1155894	Rattus norvegicus albino	dentate granule neurons	pilo-Dingledine-control-rat 5	microarray
GSM1155801	Rattus norvegicus albino	dentate granule neurons	SSSE-Wasterlain-day 1-rat 6	microarray
Data descriptors for all animals.				

**Table 2:** Transcripts in dentate granule cells with coefficient of variation <3% for expression level in the control animals (*n*=7 independent laboratories, each averaging expression over 5–6 rats)

**Gene symbol**	**group median**	**group coef. Var (%)**
Dock6	108	1.16
Ankrd13b	131	1.35
Lrba	109	1.40
Zcchc8	87	1.74
Cad	106	1.77
Zfp758	45	1.81
Timm8a1	151	1.87
Ints9 /// LOC102549712	577	1.93
Pikfyve	310	2.01
Golph3l	138	2.03
Rps6kb1	1,684	2.07
Zzef1	121	2.10
Khnyn	74	2.12
Lrfn1	462	2.15
Cryz	109	2.16
Fkbp15	126	2.19
Setd1b	107	2.19
Pacs1	152	2.24
Mlxip	214	2.27
Mtmr10	115	2.29
Strn	186	2.32
Ddx26b	1,001	2.33
Lrrc61	167	2.43
Chm	46	2.45
Stx3	106	2.47
Synm	151	2.49
Ddx52	123	2.49
Ripk1	73	2.49
LOC102549467	19	2.51
Nr2c1	44	2.60
Spin4	33	2.61
Isoc2b	92	2.63
Klf2	106	2.64
Ubfd1	133	2.66
Nol10	1,068	2.67
RGD1562608	109	2.67
Creb3l2	38	2.68
Gpr137	216	2.70
Blm	212	2.74
RGD1305422	162	2.75
Arfgef1	381	2.76
LOC102546678///Prrg3	120	2.78
Bnip1	113	2.81
LOC102552625	212	2.82
LOC100911725///Pfkfb4	69	2.83
Man1b1	229	2.86
Lepre1	111	2.88
Optc	22	2.89
LOC100912473	70	2.89
Gmds	485	2.89
Adssl1	34	2.89
Scp2d1	28	2.93
Sult2b1	97	2.94
Zbtb12	34	2.95
Casp9	105	2.95
Palm3	116	2.98
Sap30bp	224	2.98
Zfp367	32	2.99
Med27	2,190	2.99
Expression levels and SDs of genes in control condition with <3% coefficient of variation.		

**Table 3:** Transcripts in dentate granule cells with coefficient of variation >3 SDs above the mean for expression level in the control condition (*n*=7 independent laboratories, each averaging expression over 5–6 rats)

**Gene symbol**	**group median**	**group coef. Var (%)**
LOC100909504	2,944	82.9
Mis18a	238	61.9
Ttr	557	60.0
Prkcb	109	58.0
Coch	59.8	56.9
Ptgds	415	52.6
Slc17a6	195	51.9
Fam111a	59.6	50.6
Atp1a3	1,821	50.3
Dnajb6	1,310	50.0
LOC257642	1,229	49.2
Caly	2,598	48.3
Htr2c	179	47.9
Hba1///Hba2	1,331	46.6
Cartpt	649	46.5
LOC102546420	44.5	46.2
Got1	842	45.7
Adam22	1,267	45.7
Aplp1	1,636	43.9
Tbl1xr1	89.1	43.7
Smarca2	249	43.7
Ctxn1	2,175	43.5
Enc1	2,074	43.0
Psmd14	582	42.7
LOC100911806	128	42.6
Myo5b	538	42.6
Cdh13	779	42.0
LOC100911286///Pcsk1n	3,750	41.5
Slitrk3	614	41.4
Atxn2	280	41.0
Arhgap12	164	40.9
Wasl	816	40.6
Slc35g2	112	40.3
Sostdc1	60.4	40.2
Prpf40a	107	40.1
Colec12	47.0	39.8
Cacnb3	384	39.6
Pfkl	186	39.6
Tsc22d2	446	39.4
Pianp	301	39.2
Gria1	3,116	38.9
Arl8b	229	38.5
Ccnd2	1,095	38.5
Atp6v0a1	790	38.5
LOC100364062///Pkm	5,747	38.5
Brinp1	1,396	38.4
Nsg2	3,176	38.2
Enpp2	375	37.8
Slc17a7	1,062	37.7
Tanc2	144	37.7
Slitrk1	817	37.6
LOC679818	160	37.3
Pkib	596	37.0
F5	38.5	36.9
Rad17	227	36.8
Map1b	327	36.7
Mlf2	3,229	36.4
Cox5a	318	36.3
Cth	150	36.3
Lsamp	717	36.2
Ppp1r1b	950	36.2
Hpcal4	596	36.2
Myt1l	1,582	36.2
Cnih2	4,609	35.9
Tspyl2	684	35.8
Atp5a1	4,092	35.8
Nmrk1	264	35.6
Sh3bgrl	343	35.6
Tecpr1	428	35.6
Sorl1	877	35.5
Prps1	783	35.5
Atg12	191	35.5
Glyr1	1,255	35.4
RGD1564664	216	35.3
Mthfd1l	139	35.3
Pfn2	196	35.3
Dnajc5	415	35.2
Chsy3///LOC100910780	96.1	35.2
Slc12a5	9,635	35.1
Faim2	881	35.1
Cacna2d1	833	35.1
Atp6v0c	6,859	35.0
Nucb1	784	35.0
RGD1565798///Tpt1	8,075	34.8
Rcan1	161	34.8
Cck	576	34.8
Apod	210	34.8
Expression levels and SDs of genes in control condition with cv >3SD’s above mean cv.		

**Table 4:** Differentially expressed genes in common among kainate, pilocarpine and SSSE models in all 5 laboratories 1 day after SE

Aard
Abcd2
Acvr1c
Arsg
Boc
C1s
Ca10
Car11
Cbfb
Clmp
Col25a1
Crim1
Cyb561
Dclk3
Ddit4l///LOC100363484
Ddr2
Enox2
Extl1
Fam129b
Fam69c
Fat1
Gabrd
Gadl1
Gdf10
Gpc3
Htr5b
Ifitm10
Khdrbs3
Kifc3
Klf15
Klhl5
Lingo2
LOC100910632
LOC100910797
LOC100911253
LOC100912459
LOC102552294
LOC102548876
LOC102551251
LOC691995
Lox
Lrig1
Mmp9
Mpp6
Nhlh1
Nmb
Npdc1
Ntf3
Nubpl
Plk5
Podxl2
Prkacb
Prss23
Ptpn5
Ptprn
Rab26
Rbks
Rbp1
Rnasel
Rreb1
Rsph10b
Scn4b
Serinc2
Shb
Sim2
Ss18
Ssbp3
Tbc1d2b
Trh
Tut1
Wls
Zfp259
Zmiz1
